# Supplementary material for: Calculating with light using a chip-scale all-optical abacus
Source: Nat Commun. 2017 Nov 2;8:1256. doi: 10.1038/s41467-017-01506-3 (PMC5665880; doi:10.1038/s41467-017-01506-3)
Supplement: Supplementary file 1 — Supplementary Information [file 41467_2017_1506_MOESM1_ESM.pdf]

Supplementary Note 1: Measurement setup

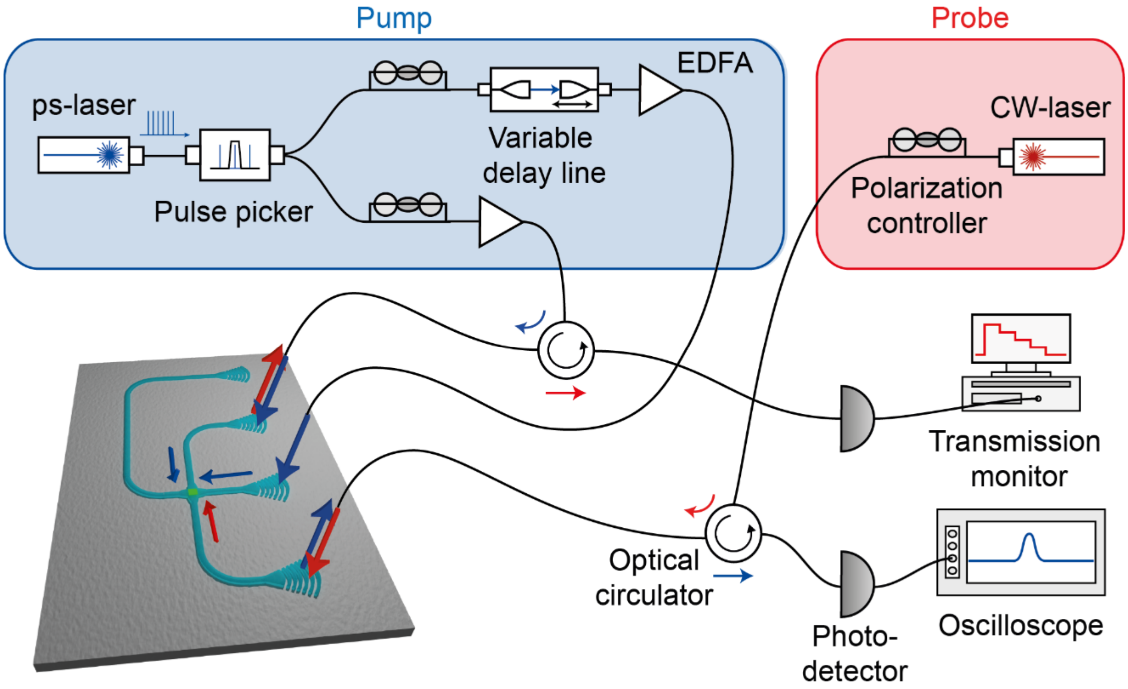

Supplementary Figure 1: Sketch of the measurement setup.
Light is coupled between the on-chip device (lower left) and the off-chip fiber-optical setup by means of focusing grating couplers. While the transmission and thus the phase-state of the PCM-cell is monitored with continuous wave probe light, picosecond optical pump pulses are used to trigger the phase-transitions within the PCM-cell. Off-chip, probe and pump light is separated from each other for separate detection by means of optical circulators.

Supplementary Note 2: Comparison between the phase change materials AIST and GST

For the experiments in the main text we used AIST for the first arithmetic examples but GST for the waveguide-crossing measurements and the subtraction example. From Supplementary Fig. 2 it can be seen that both materials show no significant difference in our all-optical applications. Supplementary Fig. 2a shows the thermo-optic behavior of GST and AIST for different pulse widths without switching. The (volatile) change in transmission is due to the change in refractive index when heating up the PCM with the optical pulse, as described in [1]. For both materials, the cooling time is faster with shorter pulse width leading to the conclusion that shorter pulses enable faster operation as the PCM-cell recovers earlier to its initial state. Also, the time-constants for both materials are on the same order of magnitude.

Looking at the write speed (Supplementary Fig. 2b) for a pulse of 10 ns length both materials again show similar properties. After the initial drop of the transmission because of the thermo-optical effect, it takes about 40 ns for both cells to settle to the new transmission level. As shown in Supplementary Fig. 2a this relaxation time is limited by thermal diffusion of the heat and can be reduced by using shorter pulses. The pulse energies used in the multilevel experiments with picosecond pulses shown in the main manuscript are for both materials in the range between 10 to 20 pJ, again implying that both phase-change materials are interchangeable when used in our PCM-cells.

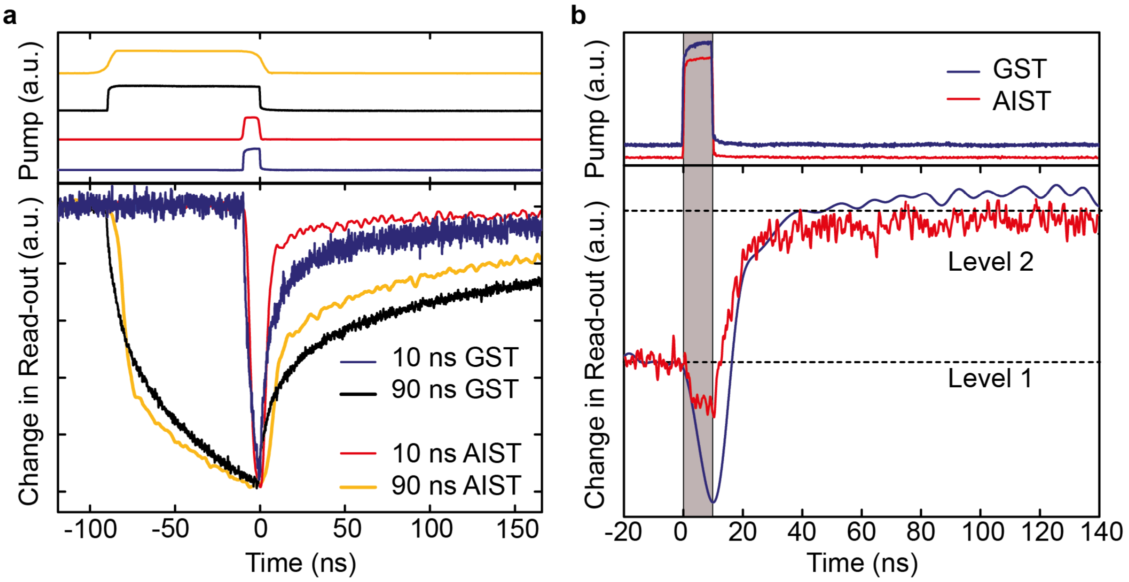

Supplementary Figure 2: Comparison of AIST and GST.

(a) Cooling time of AIST and GST for 10 ns and 90 ns pulse without switching. The initial drop is caused by the thermo-optical effect. **(b)** Write speed for a 10 ns switching pulse in a GST- and an AIST-cell. The GST-data was taken from [2].

Supplementary Note 3: Single pulse arithmetic

As mentioned in the main text we are able to operate our PCM devices with single picosecond pulses, leading to higher processing speeds and less energy consumption. Supplementary Fig. 3 shows six full switching cycles of a PCM-cell with five crystallization steps each, induced by single picosecond pulses of 8 pJ pulse energy. In order to reset the PCM to the amorphous state, five pulses (14 pJ per pulse) were used. The data reveals that all intermediate levels can be reproducibly reached with single shot switching. Further reducing the detectors noise and increasing the accuracy of the deposited power per pulse would enable operation of the PCM in higher bases, because more intermediate levels could be accessed.

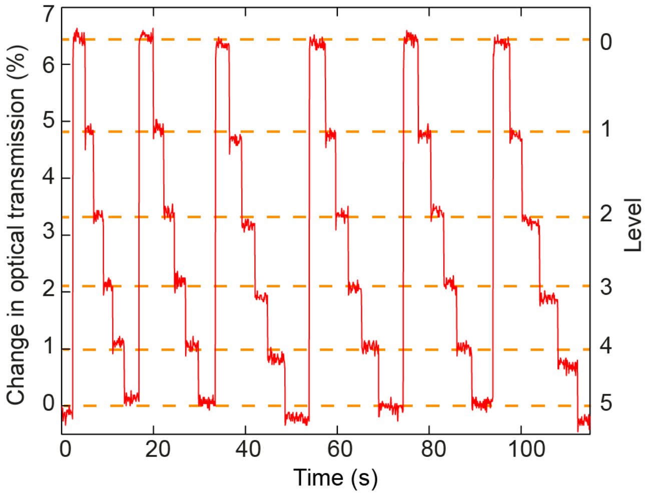

Supplementary Figure 3: Single pulse operation of PCM-cells.

Transmission measurement of a simple waveguide with a phase-change material (AIST, 7 µm length) on top. Six clearly distinguishable levels are reached with a single picosecond pulse per step. The reset pulse to go from level 5 back to level 0 consists of five pulses.

Supplementary Note 4: Arithmetic in different bases with single pulses

Figure 2 (main paper) shows basic arithmetic in base ten, yet our integrated phase-change cells are capable of operating in other bases as well, as long as the contrast between each level is clearly distinguishable and reproducible. To adjust the amount of intermediate levels and the optical contrast of a PCM-cell, it is important to control the start and end phase state of the phase-change cell, the pulse energy per step and the amount of pulses per step. For a fixed start and end state, i.e. a given percentage of the amorphous and crystalline phase of a PCM-cell, higher pulse powers or more pulses per step lead to fewer intermediate levels, because larger areas of the PCM are crystallized in a single switching step. However, in return this yields higher optical contrast between steps, which is important for reliable and repeatable calculations. Obviously, larger differences between the start and end phase state result in better overall contrast and make it easier to define more levels. Maximizing the contrast by using the whole range between fully amorphous and fully crystalline improves the reproducibility, but causes higher switching energies as phase transitions are induced in larger fractions of the PCM.

Exemplary calculations performed in different bases (five, seven and ten) using single pulses are presented in Supplementary Fig. 4. This data further illustrates the versatility of our devices, in particular since all these measurements were carried out in the same PCM-cell. The operating base is simply chosen by the number of pulses sent before resetting to the initial state. In general, if a PCM-cell is well characterized and pulse energies are chosen for operation in a certain base, it is always possible to operate the cell in a lower base by increasing the number of pulses per step. For example, doubling the number of pulses per step from one to two in Supplementary Fig. 4c would double the step sizes and reduce the base to five.

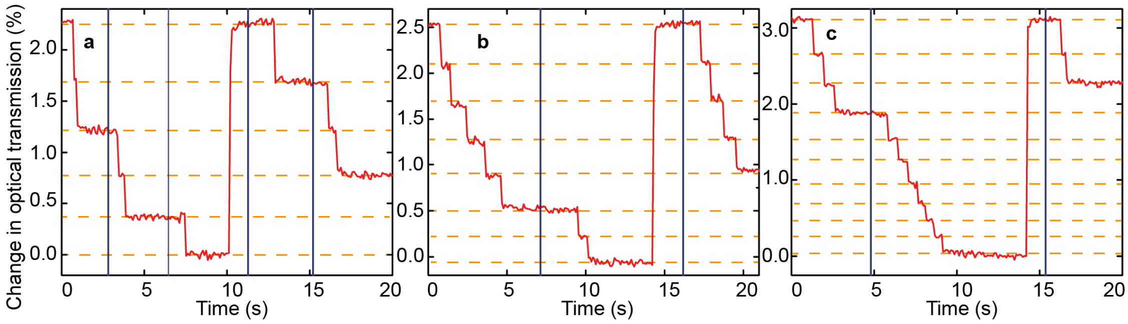

Supplementary Figure 4: All-optical operation in different arithmetic bases. **(a)** 4 x 2 calculated in base five. **(b)** 5 + 6 calculated in base seven. **(c)** 3 + 9 in base ten. All operations are carried out with the same PCM-cell (AIST, 2 μm length) and only the amount of steps before a reset was increased from (a) to (c).

Supplementary Note 5: Additional subtraction example

In addition to the main text, Supplementary Fig. 5 shows a subtraction example carried out with only one phase-change element to clarify the working principle of operating with the nine's complement. The nine's complement is obtained by subtracting the initial value from '10^N-1' with N being the number of digits. In the example shown in Supplementary Fig. 5, in order to subtract '14' from '79' we add '14' to the nine's complement of the minuend '79', which is '20'.

In the phase-change cell, as a first step '20' pulses for the minuend are applied including two carry overs. Now the subtrahend '14' is added by sending the corresponding pulses. After the third carryover, the PCM-cell is left in level four giving the result '34'. Calculating the nine's complement again leads to the correct result of the initial subtraction task, which is '65'.

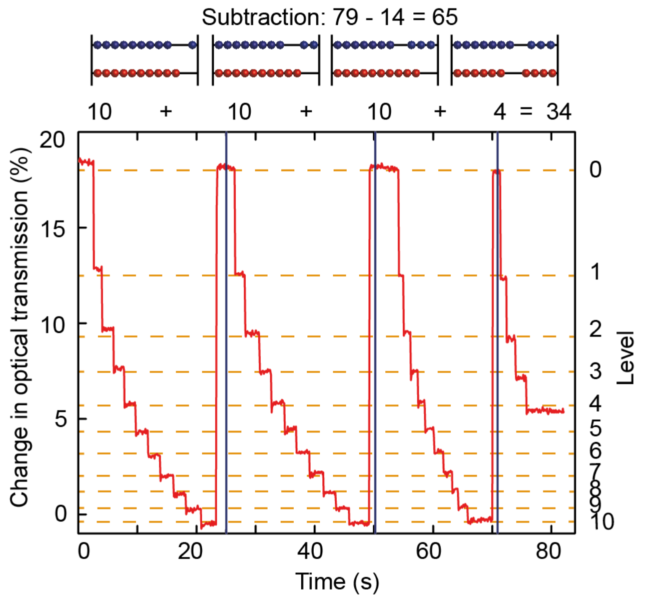

Supplementary Figure 5: Additional subtraction example.
'79-14=65' is equal to '20+14=34' if the subtrahend and the difference are considered as their nine's complement.

Supplementary Note 6: Storing the carryover

When operating phase-change cells as arithmetic units as described in the main text, the carry-over has to be stored when resetting the phase-change cell. In the example of adding 6 + 6 in Fig. 2 of the main paper, after the tenth step the PCM-cell has to be reset to the amorphous state and a carry-over has to be saved. In order to do so automatically we utilized the high power reset pulse of the first element to partially crystallize the second. Figure 3a of the main paper demonstrates how we realized this experimentally. The pulse which was formerly used to operate the first phase-change element is now split in two (off-chip, but could in future also be carried out on-chip) and guided to both PCM-cells. The splitting ratio is chosen such that a low power pulse is sufficient to induce partial crystallization in the first element while leaving the second element unaffected. A high power pulse, in contrast, resets the first element and is also intense enough to partially crystallize the second element.

Supplementary Fig. 6 shows an example measurement of a two-cell photonic abacus experimentally realized with two independent phase-change elements operated in base five. After fully crystallizing the first element in five steps, the element is reset to its initial state. By setting the splitting ratio to approximately 90:10 we ensure, that the pulse power arriving at the second element is high enough to partly crystallize it, thus inducing a first step in the transmission measurement. This procedure is repeated until the second element reaches its bottom crystallinity level. Making use of the scalability of integrated photonic structures, this basic device can be extended to more phase-change elements, capable of storing and processing higher values.

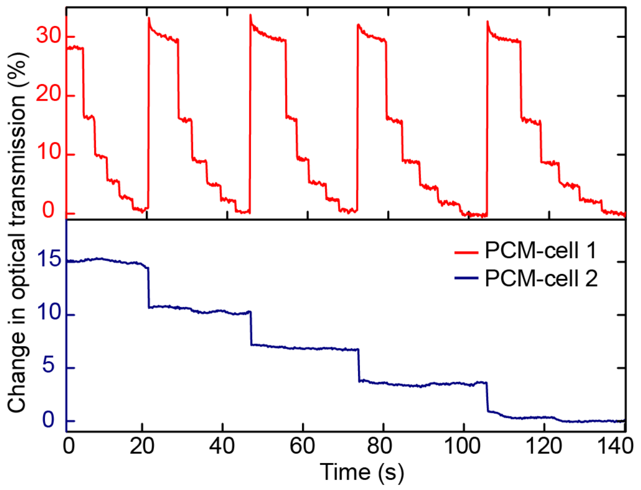

Supplementary Figure 6: Storing the carry over.

Using the reset pulse of the PCM-cell to directly store the carry-over in a second cell. During the first five crystallization pulses switching the first PCM-cell, the second cell remains unaffected. When resetting the first element to its initial state, partly crystallization is induced in the second element. The device allows therefore automatic counting up to 24 in base 5.

Supplementary Note 7: Waveguide crossing characteristics

In all two-pulse measurements we used the waveguide crossing design shown in Supplementary Fig. 7a. The four incoming waveguides are elliptically tapered from the initial waveguide width of 1.2 µm to a width of 3.0 µm at the crossing point. The length of each taper is 7.0 µm, resulting in a footprint of 17 µm x 17 µm for a single crossing. The taper is used to expand the incoming mode and therefore to reduce the angular spectrum which leads to less crosstalk. The elliptical shape of the taper is chosen to avoid excitation of higher order or radiation modes [3]. In order to experimentally determine the insertion loss of the crossing geometry, we fabricated waveguides with increasing number of crossings (Supplementary Fig. 7b) and measured the optical transmission through the devices with a continuous-wave laser at a wavelength of 1550 nm. Dividing the results by the transmission through a reference waveguide without crossings allows us to extract the loss per crossing as shown in Supplementary Fig. 7d. The slope of the linear fit reveals that this method enabled reducing the insertion loss per crossing down to (0.226 ± 0.003) dB. The crosstalk was determined with the device shown in Supplementary Fig. 7c to be less than -50 dB (measurement was limited by the detector sensitivity). Both results were also confirmed by a 3D-FDTD simulation using the Meep simulation package [4].

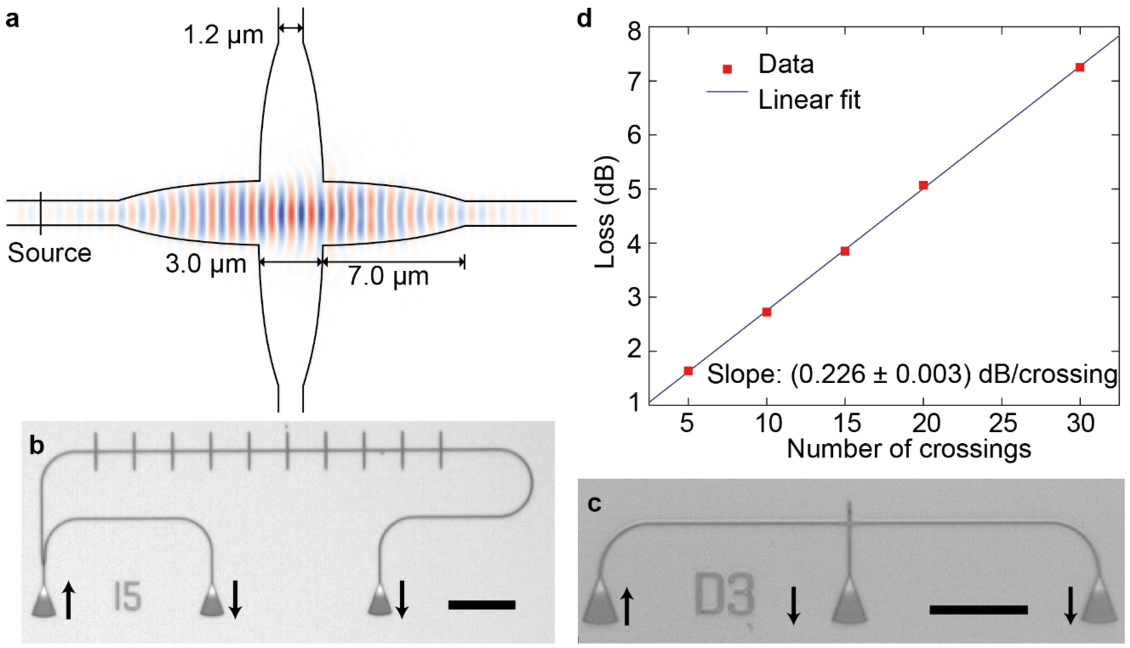

Supplementary Figure 7: Characterization of the waveguide crossings.

(a) Crossing geometry and snapshot of the electric field in plane perpendicular to the direction of propagation of a short pulse simulated via 3D-FDTD. **(b-c)** Optical micrographs of devices used to measure the insertion loss (left) and crosstalk (right). Scalebars are 100 µm. **(d)** Measured power loss as a function of the number of waveguide crossings. The slope of the fit reveals insertion loss of 0.226 dB per crossing.

A snapshot of the in-plane electric field perpendicular to the direction of propagation for a short pulse with a center wavelength of 1550 nm is shown in Supplementary Fig. 7a. The

simulated insertion loss of 0.46 dB and crosstalk of -47 dB are in good agreement with the experiment, making the crossing geometry suitable for application in larger photonic networks.

Supplementary Note 8: Switching contrast in two-pulse mode

For the two-pulse switching scheme described in the main article, $Ge_2Sb_2Te_5$ (GST)-squares of different width and 10 nm thickness were deposited on top of the waveguide crossing. A 10 nm layer of indium tin oxide (ITO) was employed as capping layer to avoid oxidation of the phase-change material. To characterize the maximum optical switching contrast the transmission through a single crossing was first measured in the amorphous (as deposited) phase and again after crystallizing the GST on a hotplate. The results are shown in Supplementary Fig. 8 and yield contrast of up to 8 dB for a completely covered crossing (9 µm²). It should be noted that these values represent the maximum contrast possible between the fully amorphous and fully crystalline state which are not entirely reached by switching with optical pulses in a waveguide.

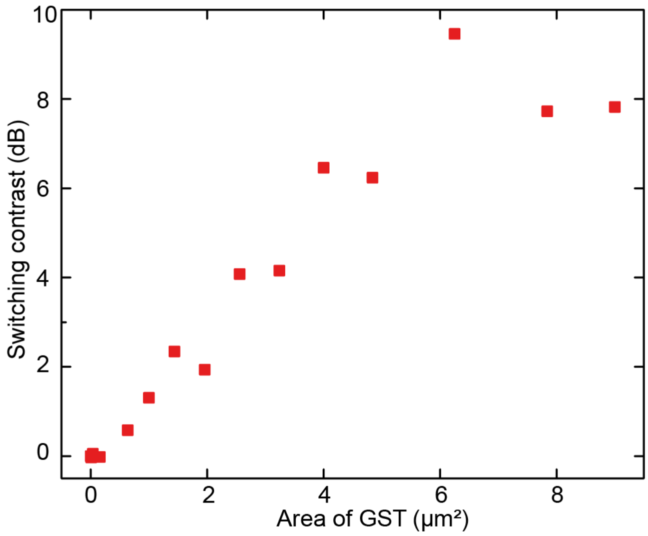

Supplementary Figure 8: **Maximum switching contrast per crossing.**

Switching contrast per crossing between the amorphous and crystalline phase as a function of the GST-cell size.

Supplementary Note 9: Mulitlevel readout in two-pulse mode

Using the waveguide crossing array does not allow for the same simple readout mechanism of the cell states in a transmission measurement which was used for readout of a single cell. Therefore, here a destructive readout scheme is applied that utilizes the change in optical transmission, when a reset pulse is send to the cell. In Supplementary Fig. 9 five distinct levels were prepared before sending a reset pulse (consisting of two overlapping pulses) to switch the PCM-cell to its initial amorphous phase ('level 0'). By measuring the change in the optical transmission (c_1 to c_4) all different levels can be distinguished enabling multilevel operation also in a waveguide crossing array.

Since a reset pulse also deletes the information that was stored in the memory element, every readout event can in principle be accompanied by a subsequent write pulse that resets the initial level again. This way the loss of information inherent to destructive readout could be overcome with only a slightly longer readout time composed of two individual pulses.

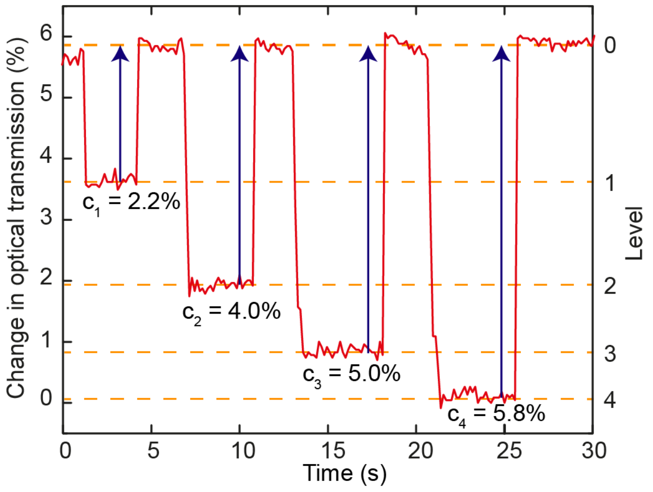

Supplementary Figure 9: Destructive multilevel readout in two pulse mode.

Multilevel scheme with five intermediate levels. The complementary switching contrast when resetting the cell from individual levels back to the top level is used to determine the phase state of a given PCM-cell. Each reset event was induced by two overlapping pulses with 21 pJ pulse energy.

Supplementary Note 10: Endurance tests

In order to investigate the repeatability of the switching process in the phase-change cells we carried out endurance cycling tests in single and two-pulse mode. A PCM-element was first taken through one million switching cycles with single pulses. During the measurements, the device transmission was monitored with an additional reference port. For amorphisation a pulse energy of 430 pJ was used and the crystallization was carried out by 18 pulses of 150 pJ energy each. The pulse width for all pulses was set to 100 ns. As can be seen in Supplementary Fig. 10a, the contrast decreases almost linearly with the number of induced switches in the PCM. However, as shown in blue in the same graph, also the reference transmission decreases to almost half of its initial value. The reduced transmission value is due to temporal drift of the measurement setup, which leads to misalignment of the input grating couplers with respect to the fiber array and thus reduced optical power in the device. The reference transmission was measured during the whole cycling test through a second independent on-chip device and is an indicator for the fraction of the initial pulse coupled into the device under test. Therefore, as the reference transmission decreases, also the pulse energies that are sent for switching the PCM-cell are reduced, leading to a reduction of the contrast over time. The lowering of the reference transmission is caused by instabilities in the experimental setup like a drift of the device. By realigning the chip in the transmission setup, the original transmission could be recovered and a switching contrast of about twenty percent was achieved again. Hence the overall contrast with respect to the pulse energy in the waveguide remains constant during the cycling tests.

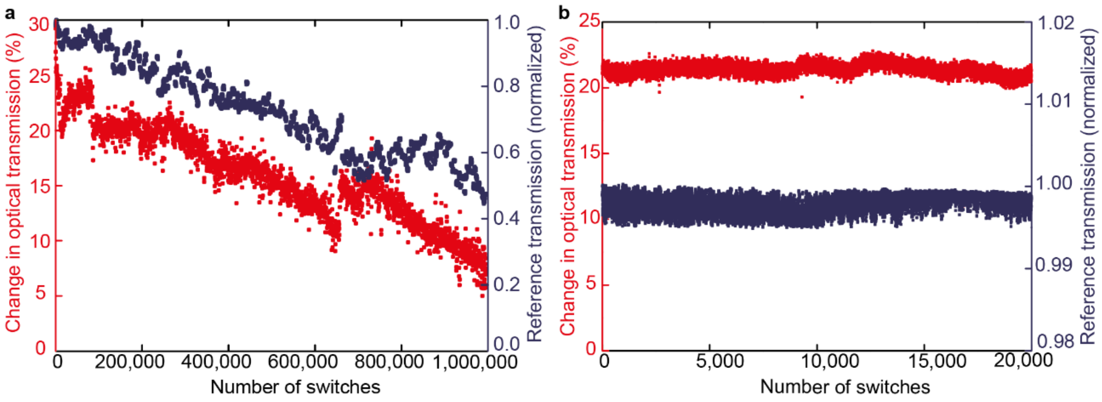

Supplementary Figure 10: Endurance of a phase change cell in single- and two-pulse mode.

(a) A phase change cell is switched a million times between amorphous and crystalline states by applying the single-pulse switching scheme. The decrease in contrast is due to a reduction of the pulse energy that is caused by device drift. **(b)** The two-pulse switching scheme was employed to switch a cell 20,000 times with a contrast of about 21%. No degradation is observed.

Supplementary Fig. 10b shows the first 20,000 switches of a PCM-cell that was cycled by applying the two-pulse addressing scheme. The average contrast stays throughout the

whole experiment at about 21% and no degradation is observed. This is also confirmed by a constant transmission level in the reference port as shown in the blue trace in Supplementary Fig. 10b. As the total pulse energies used for two-pulse switching are approximately the same (220 pJ + 240 pJ for amorphisation and 60 pJ + 80 pJ for crystallization) as in the single-pulse mode no major difference in endurance is expected compared to single pulse switching.

Supplementary References

1. Stegmaier, M., Ríos, C ., Bhaskaran, H. & Pernice, W. H. P. Thermo-optical Effect in Phase-Change Nanophotonics. *ACS Photonics* **3,** 828–835 (2016).
2. Ríos, C. *et al.* Integrated all-photonic non-volatile multi-level memory. *Nat. Photonics* **9,** 725–732 (2015).
3. Fukazawa, T., Hirano, T., Ohno, F. & Baba, T. Low Loss Intersection of Si Photonic Wire Waveguides. *Jpn. J. Appl. Phys.* **43,** 646–647 (2004).
4. Oskooi, A. F. *et al.* Meep: A flexible free-software package for electromagnetic simulations by the FDTD method. *Comput. Phys. Commun.* **181,** 687–702 (2010).
